# Effect of Information and Telephone-Guided Access to Community Support for People with Chronic Kidney Disease: Randomised Controlled Trial

**DOI:** 10.1371/journal.pone.0109135

**Published:** 2014-10-16

**Authors:** Tom Blakeman, Christian Blickem, Anne Kennedy, David Reeves, Peter Bower, Hannah Gaffney, Caroline Gardner, Victoria Lee, Praksha Jariwala, Shoba Dawson, Rahena Mossabir, Helen Brooks, Gerry Richardson, Eldon Spackman, Ivaylo Vassilev, Carolyn Chew-Graham, Anne Rogers

**Affiliations:** 1 NIHR Collaboration for Leadership in Applied Health Research (CLAHRC) Greater Manchester, Centre for Primary Care, Institute of Population Health, University of Manchester, Manchester, United Kingdom; 2 NIHR CLAHRC Wessex, Health Sciences, University of Southampton, Highfield Campus, Southampton, United Kingdom; 3 NIHR School for Primary Care Research, Centre for Primary Care, Institute of Population Health, University of Manchester, Manchester, United Kingdom; 4 Centre for Health Economics, University of York, Heslington, York, United Kingdom; 5 Primary Care & Health Services, University of Keele, Staffordshire, United Kingdom; University of St Andrews, United Kingdom

## Abstract

**Background:**

Implementation of self-management support in traditional primary care settings has proved difficult, encouraging the development of alternative models which actively link to community resources. Chronic kidney disease (CKD) is a common condition usually diagnosed in the presence of other co-morbidities. This trial aimed to determine the effectiveness of an intervention to provide information and telephone-guided access to community support versus usual care for patients with stage 3 CKD.

**Methods and Findings:**

In a pragmatic, two-arm, patient level randomised controlled trial 436 patients with a diagnosis of stage 3 CKD were recruited from 24 general practices in Greater Manchester. Patients were randomised to intervention (215) or usual care (221). Primary outcome measures were health related quality of life (EQ-5D health questionnaire), blood pressure control, and positive and active engagement in life (heiQ) at 6 months. At 6 months, mean health related quality of life was significantly higher for the intervention group (adjusted mean difference = 0.05; 95% CI = 0.01, 0.08) and blood pressure was controlled for a significantly greater proportion of patients in the intervention group (adjusted odds-ratio = 1.85; 95% CI = 1.25, 2.72). Patients did not differ significantly in positive and active engagement in life. The intervention group reported a reduction in costs compared with control.

**Conclusions:**

An intervention to provide tailored information and telephone-guided access to community resources was associated with modest but significant improvements in health related quality of life and better maintenance of blood pressure control for patients with stage 3 CKD compared with usual care. However, further research is required to identify the mechanisms of action of the intervention.

**Trial Registration:**

Controlled-Trials.com ISRCTN45433299

## Introduction

Self-management support is seen as a priority for people with long-term health problems and primary care has been identified as an important site of delivery [1] [2]. However implementation in traditional primary care settings has proved difficult, encouraging the development of alternative models [3] [4] [5] [6] [7] [8]. This, together with evidence that health-related phenomena (such as smoking cessation and weight loss) spread across social networks [9], suggests the need for a paradigm shift in how to support people with long-term health problems [1] [10].

Rather than focussing exclusively on individual factors such as self-efficacy and behaviour, self-management support might better utilize personal networks and resources within the community [11]. Self-management support undertaken within and utilising the social networks of people living with long-term conditions draws on a broader set of resources including practical and emotional support (‘illness work’) [11] [12] [13] [14]. This approach which focuses on personal communities and resources highlights the centrality of relationships in self-management, such as friends and family, and the importance of encouraging links within local communities which lie outside formal healthcare. This has the advantage of potentially building sustainable strategies for self-management over the long term, which may be of particular benefit to socially disadvantaged communities who have struggled to engage with traditional models of self-management support [15]. These populations are likely to be more amenable to self-management support options, which are more closely aligned to their everyday lives and personal preferences [11] [12] [14].

Effective management of CKD may prevent progression of the disease, and reduce the risk of cardiovascular disease [16] [17]. As emphasised by NICE guidance in the management of CKD, a key element of recommended practice is to offer high quality education at appropriate stages of the person's condition to enable understanding and informed choices about treatment [17]. It is recommended that information should be tailored to their stage and cause of CKD, any complications and the risk of progression.

However, whilst discussions around CKD may be viewed as a platform to address vascular risk and support lifestyle change, concerns have also been raised over unnecessary disease labelling, with both general practitioners and practice nurses having expressed a reticence to disclose a diagnosis of stage 3 CKD [18] [19] [20] [21] [22]. This includes a need to underplay CKD, with efforts focused on reassuring patients, particularly in older people and patients with CKD stage 3A [22]. This tension is reflected in inconsistent use of disease registers for patients who fulfil the criteria for CKD, evidence of infrequent clinical discussions about CKD, as well as low patient awareness of early stage CKD [23] [24] [25] [26]. Chronic kidney disease is also patterned by socio-economic deprivation, is usually diagnosed in the presence of other co-morbidities. CKD is therefore a good exemplar to explore the possibilities of innovative community and network focused models of self-management [27] [28] [29] [30].

This trial was conducted as part of a wider programme of work that aimed to improve health care and reduce inequalities in health for people living with chronic vascular conditions (http://clahrc-gm.nihr.ac.uk/). A key element of the programme was to improve links between different providers of support for health, including professionals, voluntary, and community resources in order to widen the options of self-management support [31]. This approach emphasises the support for people with long-term conditions outside conventional services, and the distinction between professionally-defined and patient-defined priorities in the management of long-term conditions. With a particular focus on the interface between primary care and resources in the community, the BRinging Information and Guided Help Together (BRIGHT) intervention aimed to explore the potential of network focused self-management support in the context of CKD. The BRIGHT intervention was delivered to patients with stage 3 CKD and aimed to: 1) provide patient information that incorporated both clinical and lay knowledge; 2) provide telephone-guided help by lay health workers to support patient access to community support; and 3) broaden the scope of self-management support by tailoring access to local community resources [14].

The primary aim of the trial was to test whether an intervention which provides information about self-management, tailored access to local community resources and telephone-guidance can improve health outcomes for patients with stage 3 CKD.

## Methods

The protocol for this trial and supporting CONSORT checklist are available as supporting information; see Checklist S1 and Protocol S1.

### Ethics Statement

The trial was approved by NRES Committee NorthWest Greater Manchester Central reference: 11/NW/0855. Participants provided their written consent to participate in this study.

We conducted a pragmatic, two-arm, patient level randomised controlled trial to test whether this model of self-management support is effective and cost-effective compared to usual primary care for patients with a diagnosis of stage 3 CKD. Intervention and trial design were informed by the principles of minimally disruptive medicine [32] and sought to ensure that: CKD was understood and prioritised from a patient perspective; it built on rather than disrupted existing clinical dialogue; it prioritised linkage with community resources; and recognised that CKD is a condition that is rarely diagnosed in isolation but rather needs to be discussed in the context of managing other risk factors and co-morbidities [27]. Full details of the protocol have been published elsewhere (see protocol S1) [14].

The trial was registered 5 months after recruitment of patients started, due to an error in the application of our procedures. Registration was completed during the recruitment period, and prior to follow up, analysis of the data and interpretation. Patient recruitment and follow-up took place between April 2012 and November 2012. Patients were recruited from practice CKD registers. Patients provided their written consent to participate in this study. The authors confirm that all ongoing and related trials for this intervention are registered.

### Population and setting

Eligible patients were registered with 24 general practices in Greater Manchester. This included 13 practices that participated in a renal collaborative project established by CLAHRC for Greater Manchester designed to improve identification and management of patients with CKD [33]. Patients coded with an existing clinical diagnosis of stage 3 CKD (both stages 3a and 3b, with and without proteinuria) were eligible and invited through the practice registers at GP practices. Participating general practices provided data on stage of CKD including evidence of proteinuria according to the disease classification system for CKD outlined in 2008 NICE guidance. Patients were excluded if they were unable to communicate in English, had reduced capacity to provide informed consent or were in receipt of palliative care. Only one person per household was eligible to take part to avoid potential contamination. Areas for practice recruitment were chosen because they served some of the most deprived populations of the UK: 20.4% of participants lived in the 20% most deprived local areas in England [34].

Telephone contact was made by a member of the practice team to patients due to be seen for a vascular disease review and/or if the GP or nurse prefered, through raising the study at the end of the consultation. These patients were informed about the BRIGHT trial and asked if their contact details could be forwarded to the BRIGHT team for them to be contacted for further information. If patients agreed to participate a meeting with a researcher was arranged and the first part of the questionnaire was sent.

The BRIGHT intervention was designed to align with patients' routine disease review appointments conducted by participating general practices. Informed consent and baseline data collection was carried out by a member of the research team, where possible subsequent to a patient's disease review appointment at their general practice so that we could ensure blood pressure readings were collected at or as near to baseline as possible and to ensure practices had the opportunity to discuss CKD diagnosis with patients prior to the trial. Recruitment took place between April and November 2012 and 637 eligible patients identified by practices agreed to be contacted by a researcher. The number of patients approached to be involved with the study were frequently requested by the trial team but unfortunately this information was very patchy and so it is not possible to report accurately.

### Intervention

The intervention entailed provision of a kidney information guidebook; a booklet and interactive website that tailored access to community resources; and telephone-guided help from a lay health worker. Details of the BRIGHT intervention are detailed in the published study protocol [14].

#### The kidney information guidebook

The ‘Keeping Your Kidneys Healthy’ guidebook was developed in response to findings that emerged during an initial study, which identified a need for an information resource to support patients diagnosed with stage 3 CKD [22]. This was developed with patients with stage 3 CKD and provided information based on the experiences of patients, their expressed information needs and medical evidence about treatment options. The process of development followed established principles [35] [36] [37]. The guidebook was intended to encourage patients to consider changes they could make to maintain general vascular health in the context of having a diagnosis of early stage CKD.

#### PLANS: Tailored access to community resources

The PLANS (Patient-Led Assessment for Networks Support) booklet and website have been designed to address the range of health and social problems related to living with a long-term health problem [38]. PLANS is a needs-led self-assessment tool for users to assess and prioritise their health and social needs, with links to relevant community resources and local support (collated in a database and aligned via the website to the PLANS questions). As well as offering lifestyle options (weight management classes, exercise groups, etc.), PLANS has been designed to increase social contact and promote community support awareness and engagement to sustain independent living. Patients who received the intervention were given access to the PLANS website and also given a PLANS booklet. The telephone support guided participants through the PLANS questions and options on the database.

#### Telephone facilitation from a lay health worker

The telephone facilitation model was informed by a systematic review of telephone-based self-management interventions to prevent and manage vascular disease conducted by members of the BRIGHT team [39]. Two telephone calls from a lay health worker were delivered to patients in the intervention arm; one call at one-week post-administration of the kidney information guidebook and the PLANS booklet, followed by another call four-weeks later. For the first call, a lay health worker guided patients through the PLANS booklet and website and discussed the PLANS results (e.g. local groups and resources). PLANS and the telephone support were designed to focus on patients' needs, everyday living arrangements, and personal preferences. For example, patients were asked to provide background information about themselves including how they felt they were coping with their health; living circumstances (e.g. married, family, etc); personal and social interests and current social activity. Patients were then taken through the online PLANS questionnaire by a telephone support worker to identify needs and preferences related to wellbeing, health education, adult learning, support, independent living and volunteering opportunities. Following this they were offered a set of results of local activities and services. Brief descriptions of these results were given to patients who were then asked if they were interested in any further information about any of the groups, activities or services. Hence, the intervention was tailored to meet the expressed needs of patients based on their personal preferences and the background information provided. After the first telephone call, information about the groups, activities or services were sent to the patient. For the second call, patients were asked if they had tried any of the recommended activities and were offered another opportunity to complete the PLANS questionnaire. Telephone support was available (Monday to Friday) throughout the course of the trial for unscheduled enquiries from patients.

Lay health workers participated in a three-hour training session facilitated by CB (who led the development of PLANS) about how to facilitate appropriate referrals to local resources. For the training, lay health workers received a detailed workbook and guidance on how to deliver the intervention by telephone. There were 8 telephone support workers: 3 members of staff, 4 postgraduate students, and one undergraduate student at the University of Manchester. One support worker (PJ) was employed specifically to oversee the delivery of the telephone support. Most of the support workers had a limited background in health and social care and all but one were psychology graduates.

Participants in the control arm were sent the kidney information guidebook and the PLANS booklet with links to the website at the end of the trial period. Both arms had usual access to primary care.

### Sample size

We powered the study to have 80% power to detect a standardised effect size of 0.25 between the control and intervention arms on any continuous outcome measure. This also provided 80% power to detect a change of at least 10% in the percentage of patients achieving blood pressure control. These are relatively small effects, but in line with most sizes of effect observed in our previous trials of self-management interventions [40] [41]. Basing power on an alpha level of 0.05 and a regression analysis treating outcome measures at baseline as a covariate correlated at 0.5 with follow-up (a conservative estimate), and 25% attrition of participants, we aimed to recruit between 16 and 18 general practices and a total of 500 patients across these. In the event, we recruited a higher number of practices, 24, but a slightly lower number of patients, 436, though due to a lower than anticipated attrition rate, without any loss of power.

### Randomisation

Immediately after consent and baseline data collection, an independent clinical trials unit was contacted by telephone and the participant was allocated to receive either the intervention or usual care (1∶1) via a minimisation algorithm. The minimisation procedure ensured that within each practice, as each subsequent patient was recruited the two trial arms remained well-balanced on three key prognostic factors (age, smoking status and evidence of other vascular disease). The method also includes a degree of random allocation to avoid complete determination.

### Outcomes

We collected patient level outcomes at baseline and at six months. We had three primary outcomes: positive and active and engagement in life, blood pressure control; and health related quality of life. Engagement was measured using the ‘The Positive and Active Engagement in Life’ domain of the validated Health Education Impact Questionnaire (heiQ) [42] because the item content fitted with the goal of the intervention which was to provide access to meaningful support and rewarding activities. The items are:

- I am doing interesting things in my life
- Most days I am doing some of the things I really enjoy
- I try to make the most of my life
- I have plans to do enjoyable things for myself.
- I feel like I am actively involved in life

As stated by Osborne et al. ‘this scale assesses motivation to be active and embodies the notion of participants in self-management/patient education programs engaging or re-engaging in life-fulfilling activities as a result of program involvement. Items in this construct aim to measure the individuals’ activities to convert intention into positive outcomes, and implies a change of lifestyle and life activities.’ [42]

Blood pressure was dichotomised as ‘controlled’ versus poorly controlled in accordance with 2008 NICE guidance [17] on the management of CKD for patients with stage 3 CKD with and without proteinuria (i.e. <140/90 for patients without proteinuria and <130/80 for patients with proteinuria). Blood pressure recordings were collected from general practice records; we used the readings taken closest to date of randomisation and 6 month follow up. Generic health related quality of life was measured using the EuroQoL EQ-5D index of health-related quality of life (three-level version) [43].

Secondary outcomes included: an additional five domains of the heiQ (social integration and support, skill and technique acquisition, emotional wellbeing, self-monitoring and insight, and health service navigation); the Summary of Diabetes SelfCare Activities Measure (SDSCA) [44]; the anxiety sub-scale from the Hospital Anxiety and Depression Scale (HADS-A) [45], and as a measure of CKD-specific anxiety the Emotional Response item from the Brief Illness Perception Questionnaire (BIPQ) in relation to the patient's CKD [46]; four physical and psychological well-being health education outcome measures taken from the Medical Outcomes Study (general health, social role/limitation, energy/vitality and psychological wellbeing) [47]; the UCLA Loneliness Scale [48]; the Medication Knowledge and Medication Motivation subscales from the Modified Morisky Medication Adherence Scale (MMMAS) [49]; Social capital [50] service use (frequency of contact with primary care services and hospital outpatient services); and levels of Illness, practical everyday and emotional work done by social network members (see Protocol S1). [14]

With the exception of blood pressure which was measured by a health care professional at a clinic, all outcomes were administered by post and collected by a researcher. Neither the researchers nor health care practitioners were blinded to allocation.

Service utilisation measured primary health care (GP home and practice visits, pharmacy), community health and social care, secondary health care services, out of pocket costs and costs of lost productivity. Unit cost estimates were then applied to these resource use data to generate patient level cost estimates. The cost of developing and delivering the intervention were also estimated.

### Statistical analysis

Analysis followed intention to treat principles and a pre-specified plan (see analysis plan S1). Binary outcomes (i.e. blood pressure control) were analysed using logistic regression and continuous outcomes using linear regression, using robust standard errors adjusted for the clustering of patients within practices. We controlled for baseline values of each outcome, design (i.e. minimisation) factors (age-group, smoking status, additional vascular disease) and self-report of CKD as an additional pre-specified covariate. We applied multiple imputation (MI) to baseline and 6 month variables with missing values by the chained equations approach using scores on all primary and secondary outcomes (at baseline and follow-up), CKD stage, patient demographics (age, gender, education, area deprivation, smoking status) and comorbidities. We used 20 MI sets, as examination of Monte Carlo errors indicated that this provided stability of results [51].

MI was adopted for the main analysis because this provides less biased estimates of effect than those from complete cases analysis [51] but a sensitivity analysis was conducted based on complete cases. We also conducted a further post-hoc sensitivity analysis to examine stability against two blood pressure related factors. First, the NICE criteria for blood pressure control differs between patients with and without diabetes or proteinuria; second, we found wide variation in the dates of patient routine blood pressure readings relative to dates of randomisation and 6 month follow-up [17]. To test the stability of our results to these factors we repeated the primary analysis including covariates for presence of diabetes/proteinuria and for length of time between baseline and follow-up blood pressure readings. Where either sensitivity analysis produced a result differing in significance from the primary analysis, this is indicated in the text and tables.

Analyses were conducted using STATA IC (version 12.1) with an alpha significance value of 5%. For outcomes with skewness or kurtosis ≥1.5, we used standard errors based on 1000 bootstrapped samples to derive confidence intervals and p values. We report effect sizes standardised on baseline standard deviations across all participants.

### Cost effectiveness analysis

#### Utilisation and Resource use

Resource use data and EQ5D data were collected using patient questionnaires at baseline and 6 month follow up. Missing data was imputed using multiple imputation methods. Unit cost estimates were applied to relevant resource use data to generate a total cost per patient (post-randomisation). Quality Adjusted Life Years (QALYs) were generated by applying the UK “tariff” [52] to EQ5D responses and used the Area under the Curve method with adjustment for baseline scores on the EQ5D [53]. Cost and QALY data were then synthesised to calculate an Incremental Cost Effectiveness Ratio (ICER).

## Results

**Figure 1:**
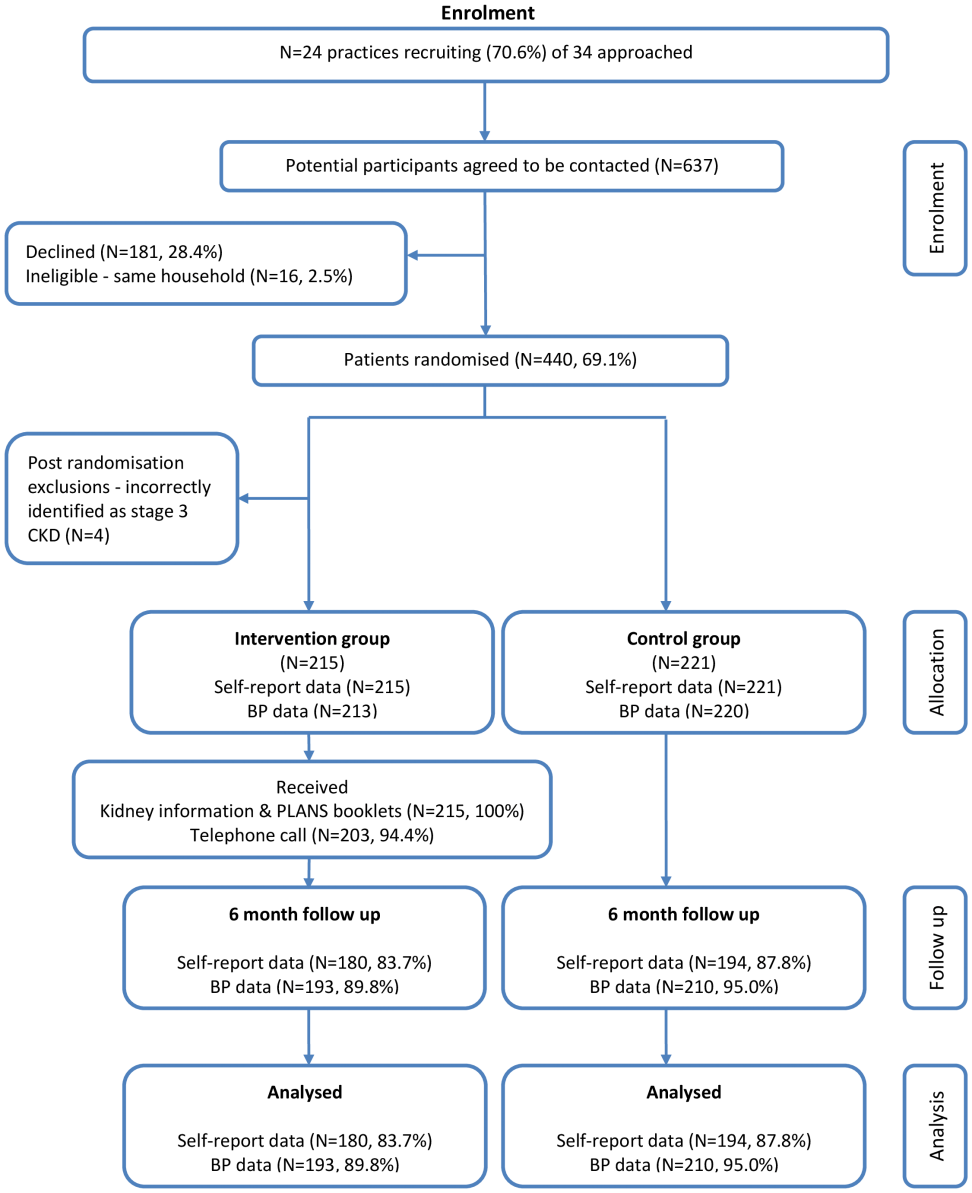
Trial CONSORT Diagram.

Figure 1 presents the trial CONSORT diagram. 34 practices were initially approached and 10 practices declined to participate in the trial prior to data collection. 440 patients were recruited from 24 GP practices (mean list size; 5815 patients) with an average of 18 patients per practice. Subsequently, 4 patients were excluded post-randomisation because they were identified as not meeting the criteria of having stage 3 CKD. Their registered GPs and the patients were informed. In total, 436 patients completed baseline data and 374 (85.7%) patients returned 6 month follow up data. We excluded two baseline blood pressure readings taken more than 15 months prior to randomisation; all other blood pressure readings were within 6 months of the target dates, with an average deviation of minus 27 days at baseline and minus 17 days at 6 month follow up.

### Baseline characteristics of study participants

58.5% (n = 255) of patients were female and few patients were non-white (1.4%). The mean age of patients was 72.1 years and 41.7% (n = 182) of patients had co-morbid established cardiovascular disease. Just over half of patients did not self-report CKD (52.1%, n = 227) (table 1).

**Table 1:** Baseline characteristics of participants.

Characteristics	BRIGHT intervention (n = 215)	Usual care (n = 221)	Total (n = 436)
**Gender:**			
Male	90 (41.9)	91 (41.2)	181 (41.5)
Female	125 (58.1)	130 (58.8)	255 (58.5)
**Mean age in years (SD):**	72.4 (9.2)	71.8 (9.0)	72.1 (9.1)
**Age group:**			
<75 years	118 (54.9)	132 (59.7)	250 (57.3)
75 years or older	97 (45.1)	89 (40.3)	186 (42.7)
**CKD Stage:**			
3a−proteinuria	156 (72.6)	156 (70.6)	312 (71.6)
3a+proteinuria	8 (3.7)	10 (4.5)	18 (4.1)
3b−proteinuria	37 (17.2)	39 (17.7)	76 (17.4)
3b+proteinuria	14 (6.5)	16 (7.2)	30 (6.9)
**Education:**			
No qualifications	104 (49.5)	84 (39.3)	188 (44.3)
1 or more O levels	18 (8.6)	19 (8.9)	37 (8.7)
1 or more A levels	3 (1.4)	10 (4.7)	13 (3.1)
NVQ/other trade/professional	64 (30.5)	90 (42.1)	154 (36.3)
Degree	21 (10.0)	11 (5.1)	32 (7.6)
**Ethnicity:**			
White	202 (98.1)	213 (99.1)	415 (98.6)
Non-white	4 (1.9)	2 (0.9)	6 (1.4)
**Mean Index of multiple deprivation (SD) (Higher score = greater deprivation):**	21.9 (15.2)	23.4 (17.5)	22.7 (16.4)
**Co-morbid established cardiovascular disease (Angina, Irregular heartbeat, Stroke, PVD, heart failure):**			
No	126 (58.6)	128 (57.9)	254 (58.3)
Yes	89 (41.4)	93 (42.1)	182 (41.7)
**Diabetes:**			
No	166 (77.2)	169 (76.5)	335 (76.8)
Yes	49 (22.8)	52 (23.5)	101 (23.2)
**No of co morbid long-term conditions (excluding kidney problems):**			
0	3 (1.4)	2 (0.9)	5 (1.2)
1–2	63 (29.3)	68 (30.8)	131 (30.1)
3–4	102 (47.4)	90 (40.7)	192 (44.0)
5–6	30 (14.0)	49 (22.2)	79 (18.1)
7+	17 (7.9)	12 (5.4)	29 (6.7)
**Smoker:**			
No	198 (92.1)	201 (91.0)	399 (91.5)
Yes	17 (7.9)	20 (9.1)	37 (8.5)
**Recruited from collaborative practice:**			
No	87 (40.5)	87 (39.4)	174 (39.9)
Yes	128 (59.5)	134 (60.6)	262 (60.1)
**Mean General Health score (SD):**	2.8 (0.9)	2.7 (1.0)	2.8 (0.9)
**Self-report CKD:**			
No	111 (51.6)	116 (52.5)	227 (52.1)
Yes	104 (48.4)	105 (47.5)	209 (47.9)
**Blood pressure control:**			
Uncontrolled	66 (31.0)	57 (25.9)	123 (28.4)
Controlled	147 (69.0)	163 (74.1)	310 (71.6)
**Mean heiQ, positive and active engagement in life (SD):**	64.6 (19.7)	65.6 (18.5)	65.1 (19.1)
**Mean EQ-5D (SD):**	0.67 (0.30)	0.67 (0.30)	0.67 (0.30)
**Mean service use in previous 6 months (SD):**	7.4 (7.1)	8.1 (14.1)	7.8 (11.2)

Values are number (percentage) unless stated otherwise.

### Engagement with intervention

The intervention comprised of the kidney information guidebook, the telephone service and the PLANS booklet and/or interactive website aimed at encouraging patients to contact and participate in local community activities or services. In summary, 82.2% (n = 147) of the 179 intervention patients who returned 6-month follow-up data reported using the ‘Keeping Your Kidneys Healthy’ guidebook at least once and 91.9% (n = 135) of these patients reported that they found it ‘useful’ or ‘very useful’. Furthermore, 87.2% (n = 156) of patients reported that the kidney information guidebook was ‘easy to read’ and 48% (n = 86) reported that the guidebook made them ‘feel less anxious’ (table 2).

**Table 2:** Intervention uptake and evaluation.

Outcome	Total intervention group 6mfu data (n = 179)
**Kidney booklet uptake**	
*How useful did you find the ‘Keeping your kidneys healthy booklet’:*	
Very useful or useful	150 (83.8)
Not very useful or useful at all	8 (4.5)
Didn't use	17 (9.5)
Missing	4 (2.2)
*How often did you refer to the ‘Keeping your kidneys healthy booklet’:*	
Used occasionally or now and then	81 (45.3)
Used only once	66 (36.9)
Haven't used	27 (15.1)
Missing	5 (2.8)
**Kidney booklet evaluation**	
*The ‘Keeping your kidneys healthy booklet’ was easy to read:*	
Strongly agree or agree	156 (87.2)
Neither agree nor disagree	11 (6.2)
Disagree or strongly disagree	1 (0.6)
Missing	11 (6.2)
*The ‘Keeping your kidneys healthy booklet’ made me feel less anxious:*	
Strongly agree or agree	86 (48.0)
Neither agree nor disagree	65 (36.3)
Disagree or strongly disagree	15 (8.4)
Missing	13 (7.3)
*The ‘Keeping your kidneys healthy booklet’ helped me discuss care with my doctor:*	
Strongly agree or agree	101 (56.4)
Neither agree nor disagree	53 (29.6)
Disagree or strongly disagree	12 (6.7)
Missing	13 (7.3)
*The ‘Keeping your kidneys healthy booklet’ helped me understand how the NHS will support my care:*	
Strongly agree or agree	117 (65.4)
Neither agree nor disagree	44 (24.6)
Disagree or strongly disagree	3 (1.7)
Missing	15 (8.4)
*The ‘Keeping your kidneys healthy booklet’ enabled you to cope with life:*	
Much better or better	70 (39.1)
Same or less	74 (41.3)
Missing/not applicable	35 (19.6)
*The ‘Keeping your kidneys healthy booklet’ enabled you to understand your condition:*	
Much better or better	93 (52.0)
Same or less	56 (31.3)
Missing/not applicable	30 (16.8)
*The ‘Keeping your kidneys healthy booklet’ enabled you to cope with your condition:*	
Much better or better	80 (44.7)
Same or less	63 (35.2)
Missing/not applicable	36 (20.1)
*The ‘Keeping your kidneys healthy booklet’ enabled you to keep yourself healthy:*	
Much better or better	93 (52.0)
Same or less	57 (31.8)
Missing/NA	29 (16.2)
*The ‘Keeping your kidneys healthy booklet’ enabled you to feel confident about your health:*	
Much more or more	69 (38.6)
Same or less	79 (44.1)
Missing/NA	31 (17.3)
*The ‘Keeping your kidneys healthy booklet’ enabled you to help yourself:*	
Much more or more	74 (41.3)
Same or less	72 (40.2)
Missing/NA	33 (18.4)
**Telephone support call uptake:**	
*How useful did you find the telephone support call:*	
Very useful or useful	111 (62.0)
Not very useful or useful at all	14 (7.8)
Didn't use	47 (26.3)
Missing	7 (3.9)
**Telephone support call evaluation:**	
*I would like to use this type of service again:*	
Strongly agree or agree	47 (26.3)
Neither agree nor disagree	86 (48.0)
Disagree or strongly disagree	29 (16.2)
Missing	17 (9.5)
*I felt the conversation with the telephone support worker was relevant to me:*	
Strongly agree or agree	91 (50.8)
Neither agree nor disagree	59 (33.0)
Disagree or strongly disagree	15 (8.4)
Missing	14 (7.8)
*I felt the conversation with the telephone support worker understood my needs and concerns:*	
Strongly agree or agree	99 (55.3)
Neither agree nor disagree	58 (32.4)
Disagree or strongly disagree	7 (3.9)
Missing	15 (8.4)
*The conversation encouraged me to think about trying local activities:*	
Strongly agree or agree	72 (40.2)
Neither agree nor disagree	64 (35.8)
Disagree or strongly disagree	28 (15.7)
Missing	15 (8.4)
*The conversation was easy to follow:*	
Strongly agree or agree	131 (73.2)
Neither agree nor disagree	28 (15.6)
Disagree or strongly disagree	4 (2.2)
Missing	16 (8.9)
**Website uptake:**	
*How useful did you find the website:*	
Very useful or useful	41 (22.9)
Not very useful or useful at all	14 (7.8)
Didn't use	108 (60.3)
Missing/not applicable	16 (8.9)
*How many times did you visit the website?:*	
Used occasionally or now and then	18 (10.1)
Used only once	29 (16.2)
Haven't used	90 (50.3)
Missing/not applicable	42 (23.5)
**Website evaluation:**	
*I found the website easy to use:*	
Strongly agree or agree	26 (14.5)
Neither agree nor disagree	52 (29.1)
Disagree or strongly disagree	12 (6.7)
Missing/not applicable	89 (49.7)
*I found the website relevant to me:*	
Strongly agree or agree	14 (7.8)
Neither agree nor disagree	62 (34.6)
Disagree or strongly disagree	13 (7.3)
Missing/not applicable	90 (50.3)
**PLANS booklet uptake:**	
*How useful did you find the PLANS booklet:*	
Very useful or useful	119 (66.5)
Not very useful or useful at all	29 (16.2)
Didn't use	26 (14.5)
Missing	5 (2.8)
**PLANS groups uptake:**	
*Total number of PLANS groups patient's contacted:*	
0	101 (56.1)
1–2	34 (18.9)
3+	6 (3.4)
Missing	6 (3.3)
Not applicable - No group information requested	33 (18.3)
*Total number of PLANS groups patient's attended:*	
0	108 (60.0)
1	19 (10.6)
2	6 (3.3)
3	3 (1.7)
Missing	11 (6.1)
Not applicable - No group information requested	33 (18.3)
*Total number of PLANS groups patient's currently attending:*	
0	122 (67.8)
1	10 (5.6)
2	2 (1.1)
3	2 (1.1)
Missing	11 (6.1)
Not applicable - No group information requested	33 (18.3)

Values are number (percentage).

Overall, 94.4% (n = 203) of patients in the intervention arm received an initial telephone support call and 97.5% (n = 198) of these received a follow up support call 4 weeks later. 62% (n = 111) of patients reported that they found the telephone support call ‘useful’ or ‘very useful’ and 40.2% (n = 72) stated that the call encouraged them to ‘think about trying local activities’.

Around a quarter of patients reported accessing the website (26.3%, n = 47) and 66.5% (n = 119) reported that the PLANS booklet was ‘useful’ or ‘very useful’. Forty (22.3%) patients contacted at least one PLANS group, 28 (15.6%) attended at least one PLANS group and 14 (7.8%) reported that they were currently attending at least one PLANS group at 6-month follow-up.

### Analysis of outcomes

In analyses of primary outcomes, blood pressure was within NICE guidance for a significantly greater proportion of patients in the intervention group at 6 months, relative to baseline (adjusted odds-ratio = 1.85; 95% CI = 1.25, 2.72) (table 3). Mean health related quality of life was also significantly higher for the intervention group (adjusted mean difference = 0.05; 95% CI = 0.01, 0.08). However, patients in the intervention group did not differ significantly on positive and active engagement in life [42]. These results were confirmed by a sensitivity analysis using complete cases and a further post-hoc analysis adjusted for differing sub-group criteria for blood pressure control and differing times of measurement.

**Table 3:** Results of regression analyses of primary and secondary outcomes at 6 months.

	Unadjusted mean (SD); N					
Outcome	BRIGHT intervention^1^	Usual care^1^	Adjusted mean difference^2^ (95% CI)	P Value^2^	Effect size^2^	Effect size from complete cases analysis^1^
**Primary Outcomes**						
Blood pressure control	67.3%^3^; 193	55.3%^3^; 210	1.85^4^ (1.25, 2.72)	0.002*	1.85^4^ (1.25, 2.72)	1.90^4^ (1.28, 2.83)
Positive and active engagement with life (heiQ) positive and active engagement in life *Higher score = higher engagement with life*	66.4 (19.7); 180	66.5 (17.6); 194	0.00 (−3.20, 3.21)	0.999	0.00 (−0.16, 0.16)	−0.01 (−0.17, 0.16)
Health-related Quality of Life (EQ-5D)	0.71 (0.28); 179	0.67 (0.29); 193	0.05 (0.01, 0.08)	0.027*	0.16 (0.03, 0.29)	0.18 (0.06, 0.30)
**Secondary Outcomes**						
heiQ, social integration and support *Higher score = Higher social integration*	69.6 (20.3); 178	69.4 (15.6); 193	0.78 (−1.70, 3.26)	0.537¶	0.05 (−0.10, 0.20)	0.01 (−0.16, 0.19)
heiQ, skills and technique acquisition *Higher score = Higher skills and technique acquisition*	65.4 (14.6); 177	65.0(13.1); 192	1.49 (−0.88, 3.85)	0.218¶	0.10 (−0.06, 0.27)	0.13 (−0.05, 0.31)
heiQ, emotional wellbeing *Higher score = Higher negative affect*	31.4 (22.2); 180	34.0 (22.2); 194	−1.85 (−5.75, 2.05)	0.329	−0.09 (−0.25, 0.07)	−0.09 (−0.25, 0.07)
heiQ, self-monitoring and insight *Higher score = Higher self monitoring and insight*	70.7 (12.2); 180	70.7 (11.5); 194	0.47 (−1.65, 2.58)	0.644	0.04 (−0.11, 0.20)	0.03 (−0.13, 0.18)
heiQ, health services navigation *Higher score = higher health service navigation*	70.5 (16.2); 179	69.4 (15.9); 193	1.88 (−1.28, 5.04)	0.226	0.14 (−0.06, 0.34)	0.12 (−0.08, 0.32)
SelfCare Activities (SDSCA) *Higher score = higher self-care*	4.5 (1.2); 172	4.2 (1.2); 191	0.23 (0.04, 0.41)	0.019*	0.20 (0.06, 0.34)	0.17 (0.05, 0.29)
Anxiety (HADS-A) *Higher score = greater anxiety*	4.6 (3.7); 179	5.2 (4.1); 194	−0.51 (−1.05, 0.02)	0.060	−0.14 (−0.28, 0.01)	−0.14 (−0.27, 0.00)
CKD-specific anxiety (BIPQ Emotional Response item asked in relation to CKD) (6 months only) *Higher score = greater anxiety*	1.2 (2.0); 179	1.6 (2.2); 190	−0.46 (−0.97, 0.05)	0.073	−0.21 (−0.42, 0.00)	−0.18 (−0.39, 0.02)
MOS, General Health *Higher score = Better general health*	2.8 (1.0); 179	2.8 (0.9); 193	0.01 (−0.12, 0.14)	0.832	0.01 (−0.12, 0.14)	−0.02 (−0.14, 0.10)
MOS, Social/Role Activities Limitations *Higher score = LOWER social limitations*	73.2 (28.2); 177	68.7 (30.5); 194	1.85 (−3.68, 7.37)	0.492	0.06 (−0.10, 0.22)	0.05 (−0.13, 0.22)
MOS, Energy/Vitality *Higher score = higher energy and vitality*	52.4 (22.0); 179	50.8 (21.8); 194	2.77 (−0.39, 5.93)	0.082	0.13 (0.00, 0.26)	0.16 (0.04, 0.28)
MOS, Psychological Wellbeing *Higher score = higher psychological wellbeing*	74.7 (18.8); 179	74.0 (19.9); 193	1.28 (−1.18, 3.73)	0.286	0.06 (−0.06, 0.18)	0.08 (−0.04, 0.19)
Loneliness (UCLA Loneliness Scale) *Higher score = LOWER loneliness.*	30.3 (5.3); 177	31.0 (4.4); 192	−0.06 (−0.82, 0.69)	0.861	−0.01 (−0.15, 0.14)	−0.01 (−0.15, 0.14)
MMMAS, Medication knowledge (6 months only) *Higher score = higher medication knowledge*	2.6 (0.6); 175	2.6 (0.6); 191	−0.05 (−0.16, 0.05)	0.331¶	−0.09 (−0.26, 0.09)	−0.07 (−0.26, 0.12)
MMMAS, Medication motivation (6 months only) *Higher score = higher medication motivation*	2.7 (0.6); 177	2.7 (0.5); 192	−0.03 (−0.13, 0.07)	0.568¶	−0.06 (−0.25, 0.14)	−0.05 (−0.25, 0.16)
Social capital: Health Survey for England *Higher score = greater satisfaction with opportunities to participate in the community*	3.7 (0.8); 178	3.6 (0.8); 188	0.08 (−0.08, 0.23)	0.325	0.09 (−0.08, 0.26)	0.09 (−0.08, 0.25)
Service use	6.1 (5.5); 180	6.5 (4.7); 194	−0.30 (−1.10, 0.49)	0.455¶	−0.03 (−0.10, 0.04)	−0.03 (−0.1, 0.04)
Social network – Illness work *Higher score = greater help with illness work from social network*	10.3 (8.4); 160	11.5 (9.0); 182	−1.18 (−3.02, 0.66)	0.208¶	−0.21 (−0.54, 0.12)	−0.24 (−0.61, 0.13)
Social network – Practical work *Higher score = greater help with practical work from social network*	6.2 (6.2); 159	8.1 (7.1); 176	−2.02 (−3.68, −0.36)	0.017* ¶	−0.46 (−0.83, −0.08)	−0.53 (−0.93, −0.12)
Social network – Emotional work *Higher score = greater help with emotional work from social network*	13.4 (10.4); 163	14.9 (11.4); 182	−0.67 (−2.47, 1.12)	0.463¶	−0.09 (−0.31, 0.14)	−0.13 (−0.35, 0.09)

1 Values from complete cases.

2 Values from multiple imputation analysis.

3 Percentage of patients with controlled blood pressure.

4 Adjusted odds ratio.

¶ P value based on boot strapped estimate of variance.

*Significant at p<0.05.

**Figure 2:**
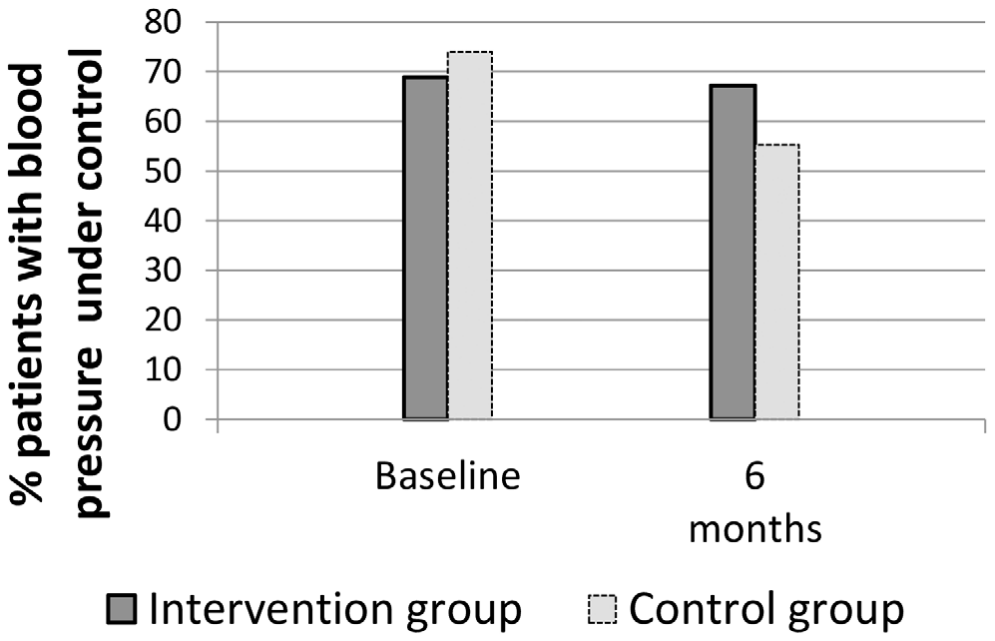
Percentages of intervention and control patients with controlled blood pressure.

**Figure 3:**
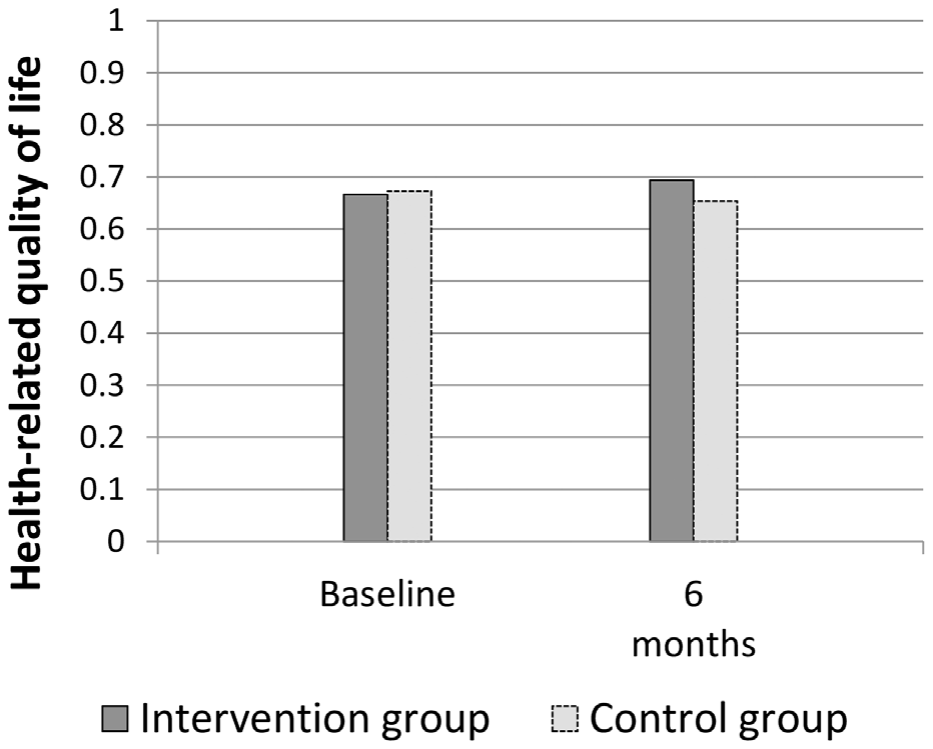
Health related quality of life for intervention and control patients.

Figure 2 depicts the unadjusted rates of blood pressure control for the two groups, across time. The rate of control remained stable in the intervention group over the 6 months, but dropped very considerably in control patients, suggesting that the effect of the intervention was to help patients maintain, but not increase, blood pressure control. Mean systolic and diastolic blood pressure in intervention patients changed very little over the 6 months (from 131.6 to 131.5 and 73.6 to 73.4 respectively), but increased in control patients (129.0 to 135.2 and 73.4 to 74.5 respectively). For health related quality of life however, there was an increase in mean score for intervention patients, compared to no change in the control group mean (Figure 3).

Analysis of secondary outcomes (table 3) indicated that patients in the intervention group reported significantly higher levels of self-care activity at 6 months compared to control patients (adjusted mean difference = 0.23; 95% CI = 0.04, 0.41). In contrast to our hypothesis, patients in the intervention group experienced a significant reduction in practical work undertaken by their social network at 6 months (adjusted mean difference = −2.02; 95% CI = −3.68, −0.36). All results remained unchanged under both sensitivity analyses, with the exception of a significant (p<0.05) increase in energy/vitality scores for intervention patients under complete cases analysis.

#### Cost effectiveness

Based on a multiple imputation analysis, there were statistically significant differences between the groups in terms of Quality Adjusted Life Years generated over the 6 month time horizon of the trial. The absolute difference, in favour of the BRIGHT intervention, of 0.012 QALYs (95%CI 0.002 to 0.022) after adjustment including baseline EQ5D scores would be considered statistically significant at conventional levels. Again based on multiple imputation data, the intervention group was associated with a reduction in costs compared with control due to lower secondary care attendance. The mean difference in total cost was £175 (95%CI −£284 to £635). Control groups costs were inflated by two individuals spending 60+ days as inpatients. However, even when these are removed, the intervention is associated with reduced costs (mean difference £123, 95% CI −£103 to £349). Results from the multiple imputation were consistent with the complete case analysis, where again QALYs were significantly higher in BRIGHT group and costs were reduced.

## Discussion

### Principal findings of the study

The BRIGHT intervention used tailored information and telephone-facilitated help to enhance self-management by signposting patients to community resources. The intervention was associated with modest but significant improvements in health related quality of life and better blood pressure control, which was maintained in the intervention arm but not in the control arm. However, with the exception of a small increase in self-care activities, these effects were not matched by improvements in patient-reported positive and active engagement in life.

These modest but significant benefits occurred in the context of relatively limited engagement with some aspects of the interventions. Relatively few patients in the intervention arm of the trial reported engagement with a PLANS group, with only 7.8% reporting attending at least one PLANS group at 6-months. This data might suggest that the PLANS component was ineffective and could be omitted from the intervention. We argue that would be premature. It is difficult to remove individual components from a complex intervention without potentially threatening the effectiveness of the whole. Qualitative work conducted as part of the BRIGHT trial [54] shows that the PLANS and telephone-guided help components of the intervention were more challenging for patients, and also provided some evidence that the process encouraged positive action by giving patients the opportunity to reflect on their current circumstances. A better focus may be on enhancing the intervention, by increasing the number of follow up consultations, or providing supported access to groups (such as transport). However, lack of clarity about the causal mechanisms suggests that further work is needed to explore these issues before more widespread commissioning can be recommended.

### Strengths and limitations of this study

The intervention is a generic model that could be adapted for any chronic condition, but CKD was selected because of its association with socioeconomic deprivation as well as controversies over the relevance of the condition to patients and practitioners. The majority of patients with stage 3 CKD are managed in primary care and though effective self-management support is highlighted as a key aspect of care for this practice population [17], thus far there has been limited research and evidence on how to achieve this [55]. Intervention and study design was informed by the principles of minimally disruptive medicine [32] in order to support the embedding and integration of recruitment for the BRIGHT intervention with primary care procedures. We achieved high levels of participation by general practices. Respecting GP judgement on eligibility, 440 out of 637 (69%) potential participants agreed to take part in the trial. In addition, participant self-report indicated that the components of the intervention were both acceptable and useful.

We recognise that the provision of information may also be relevant to patients with CKD stages 1 or 2 with proteinuria, who are also at increased cardiovascular risk [16]. However, for trial purposes, in order to be confident of recruiting patients with a recorded diagnosis, we limited recruitment to patients with stage 3 CKD - a stage incentivised by the Quality and Outcomes Framework of the new General Medical Services contract [56]. Participation was restricted to patients able to read and understand English so we cannot assume that the findings of this study would be similar for patients in whom English is not their first language. None of the staff who delivered the intervention had clinical training (though one was a member of the research team and all but one were psychology graduates), and the BRIGHT training was brief. If the intervention was to be rolled out, it is likely that it could be delivered by a range of staff, including staff in voluntary groups, more established lay health workers such as health trainers, or telecare staff.

Limitations of the study are that a longer follow-up would have been preferable to show if the improvements could be sustained. The trial used validated instruments to measure health related quality of life and positive and active engagement in life [42] [52], though we recognise there is potential for bias in patient self-report outcomes in an unblinded trial. Resource use was also collected using patient questionnaires at baseline and follow up, which is a potential limitation as self-report may not always agree with sources such as service records. Blood pressure measurement was based on routine data collection rather than formal research assessments, and there was also a small difference in retention rates between arms. However, we used robust allocation concealment, achieved high levels of retention overall, and used appropriate imputation to account for missing data, and have no reason to doubt that the blood pressure outcomes are valid. Nevertheless, caution is required with interpretation of the blood pressure outcome, since the mechanism of action remains unclear. There were no significant differences between the two arms of the study in terms of positive and active engagement in life (primary outcome), GP and nurse visits, or medication adherence (secondary outcomes). We did not collect data on prescribing of antihypertensive medication, which is a potential limitation in understanding mechanisms of action though an effect was observed on self-management activities (secondary outcomes) using a validated measure that assesses diet, exercise and smoking behaviour. The intervention was intended to facilitate greater engagement with community groups and resources, but contrary to our original hypothesis, in addition to no significant effect in positive and active engagement in life, levels of support from personal social networks (for practical work) were significantly reduced in the intervention arm. One plausible explanation is that intervention patients felt enabled to do more for themselves, thus reducing dependence on others: high dependency has negative emotional effects and greater autonomy is a desired objective of many people with limiting health problems [57]. A better understanding of the mechanisms by which these innovative network interventions achieve their effects is a key research question for the future.

### Comparison with other studies and key implications of the research

The BRIGHT trial aimed to connect people to resources in order to support vascular health and broader wellbeing, and builds on growing evidence that diverse and supportive networks can improve health [9]. This approach values support provided outside healthcare settings, though it may be important that such support is legitimised by a GP or nurse [58]. Our intervention may have created a critical moment or tipping point for patients. Perhaps this method of increasing awareness of CKD gave control to the patient enabling them to initiate responses or actions.

A recent meta-analysis highlighted the importance of blood pressure lowering as an effective strategy for reducing cardiovascular events in people with CKD [59]. Our results demonstrated deterioration in blood pressure control in usual care patients and maintenance of control in those receiving the intervention, but the comparative benefits were similar in magnitude to structured care in general practice for patients with poorly controlled hypertension [60].

There have been concerns among GPs and nurses that raising awareness of CKD could have detrimental effects on patients because of raised anxiety [22], but the current study provides a model for informing patients about the diagnosis in the context of the maintenance of general vascular health, with good evidence for benefits in both clinical and quality of life outcomes and no noticeable increase in anxiety. Broadening and personalising discussions around kidney health may address fears over disease labelling and support the practise of effective minimally disruptive medicine [21] [32]. In light of recent clinical guidance, in addition to the BRIGHT trial's focus on placing CKD in the context of general vascular health, patient and carer involvement in the development of information resources to support the prevention and management of acute kidney injury (AKI) may also be warranted [61]. Elderly housebound patients living with complex co-morbidities are particularly at risk of AKI - a syndrome that is common, harmful, costly and potentially avoidable [62].

These findings provide support for an effective and cost-effective strategy to improve outcomes for people with CKD and other vascular diseases, which takes health management into everyday contexts. Moving away from traditional individually-focused models of self-management allows for appreciation of the everyday challenges faced by people with long-term conditions and draws attention towards potentially valuable forms of non-clinical support [12].

## Conclusions

Findings that an intervention delivered outside the health encounter can have a significant (albeit modest) impact on health outcomes and can be cost effective demonstrate the merit of a community focussed approach to self-management support. The BRIGHT trial highlights the potential importance of widening the types of support offered to people with long-term conditions, in particular by shifting the emphasis of self-management support towards community resources and personal networks of support. Although further work on mechanisms of effect is clearly warranted, these findings, alongside evidence that interventions to change professional behaviour to support self-management are of limited benefit, suggest that better outcomes may be achieved by placing more effort and resources in communities and that more research should be directed towards determining ways and means of embedding health management activities into everyday life.

## Supporting Information

Analysis Plan S1 **Trial analysis plan.**
(DOC)

Checklist S1 **CONSORT Checklist.**
(DOC)

Protocol S1 **Trial Protocol.**
(PDF)
