# Supplementary material for: Effect of Information and Telephone-Guided Access to Community Support for People with Chronic Kidney Disease: Randomised Controlled Trial
Source: PLoS One. 2014 Oct 16;9(10):e109135. doi: 10.1371/journal.pone.0109135 (PMC4199782; doi:10.1371/journal.pone.0109135)
Supplement: Analysis Plan S1 — Trial analysis plan. (DOC) [file pone.0109135.s001.doc]

**BRIGHT ANALYSIS PLAN**

27^th^ September 2013

This document specifies the planned statistical analysis of the BRIGHT RCT to evaluate the effectiveness of the BRIGHT intervention.

**Type of study**

The trial is a two-arm randomised controlled trial (RCT). Randomisation was at the level of the patient. Data was collected at baseline and 6 months.

**Special issues**

Around half of patients (52.1%) did not self-report kidney problems despite being identified from a register as having stage 3 CKD by practices.

Some patients had their blood pressure (BP) taken more than 8 weeks (>56 days) prior to randomisation (n=38, 8.7%) or more than 8 weeks post randomisation (n=1, 0.2%).

At 6 month follow up, some patients had their BP taken more than 8 weeks (>56 days) prior to completing the follow up questionnaire (n=26, 6%) or more than 8 weeks after returning the follow up questionnaire (n=1, 0.2%).

**Objectives**

Main analysis

The main analysis will test for a treatment effect on each primary and secondary outcome variable.

## Sample

For each outcome, the sample will be all patients who returned a baseline and/or 6 month follow-up questionnaire. The primary analysis will use multiple imputation to substitute missing values at baseline and/or follow-up. A sensitivity analysis on this will be a complete cases analysis that excludes patients with missing data.

**Analysis Populations**

Intention to treat.

**General issues of analysis**

Outcomes measured using ordinal scales will be treated as continuous variables for the purposes of analysis. The sample size is large enough to justify this. However, results for non-normal variables (skew or kurtosis >1.5) will be confirmed using bootstrap analysis. Dichotomous outcomes will be analysed using logit models.

The main test of the intervention will be the test that the overall main effect of the intervention is zero.

Baseline values of outcomes, design factors and awareness of CKD at baseline will be included in all analyses. The design factors are those used in the minimisation procedure for allocating patients to trial arms: age-group; smoking status; and additional vascular disease.

**1. Main Analyses of Primary Outcomes**

Research hypotheses to be tested.

There are no overall significant differences in the primary outcomes between intervention and control groups at 6 months.

| **Primary Outcome (all at 6 months)** | **Description** |
| --- | --- |
| Blood pressure control | Computed as a dichotomy (controlled/uncontrolled) based upon the NICE guidelines: <140/90 for patients without proteinuria and <130/80 for patients with proteinuria). (0) Uncontrolled (1) Controlled. |
| Self-management ability (HeiQ – positive and active engagement in life) | Score on the ‘positive and active engagement in life’ subscale from the HeiQ (Health education and impact questionnaire) scale, computed as the mean across the 5 items, rescaled to the range 0-100.  Items D1, D4, D7, D8, D12. |
| Health-related Quality of Life | The HRQOL utility score derived from the ED-5D  Items A1 – A5 |

**Statistical methods to be employed**

Test of the intervention: The overall effect of the intervention will be tested by the main effect of treatment group from the regression analysis.

Analysis method

Two-level (patients within practices) multivariate regression. Baseline data was collected for all the primary outcomes. Logistic regression will be use for the binary outcome of blood pressure and linear regression will be used for continuous outcomes (positive and active engagement in life and EQ-5D).

Dependent variable: Primary outcome at 6 months

Independent variables: treatment group, awareness of CKD at baseline, baseline value of primary outcome, design factors (age-group, smoking status and additional vascular disease). The analysis will also control for the clustering of patients within GP practices.

Level of significance: 0.05

Alternative tests if distribution assumptions are violated

Regression with bootstrapping.

Adjustments of significance and confidence levels due to multiplicity of outcomes

Each primary outcome addresses a different hypothesis and therefore adjustment for multiple testing will not be applied.

# Sensitivity analysis

See relevant section below

**2. Analyses of Secondary Outcomes**

Research hypotheses to be tested.

There are no significant differences in the secondary outcomes between intervention and control groups.

| **Secondary Outcome** | **Description** |
| --- | --- |
| **Outcomes at 6 months** | |
| Self management (heiQ) | Computed as the mean across the items for each subscale, rescaled to the range 0-100. Items E1 – E31. |
| Social integration and support |
| Skills and technique acquisition |
| Emotional wellbeing |
| Self-monitoring and insight |
| Health services navigation |
| Self-care activity | Summary of Diabetes Self-Care Activities (SDSCA). Computed as the sum of items F1 – F7 rescaled to the range 0 -7. |
| General anxiety | Anxiety subscale from the Hospital Anxiety and Depression Scale (HADS-A). Computed as the sum of items G1-G7. |
| CKD specific anxiety (6mfu only) | One item - C2, ‘How much do your Kidney Problems (CKD) affect you emotionally? With a range of 0 (not affected at all) – 10 (extremely affected). |
| General Health | Question C1  Treated as a continuous outcome. Five levels, recoded as (1) Poor (2) Fair (3) Good (4) Very good and (5) Excellent. |
| Social/Role Activities Limitations score | Scale constructed from Questions C2-C5 as documented by Lorig  Overall score scaled to range from 0-100. |
| Energy/Vitality score | Scale constructed from Questions C6-C10 as documented by Lorig.  Overall score is scaled to range from 0 to 100 |
| Psychological Wellbeing | Scale constructed from Questions G1-G5 as documented by Lorig  Overall score scaled to range from 0-100 |
| Loneliness | UCLA Loneliness Scale items J1 – J9. Scores range from 9 (very lonely) to 36 (Not lonely at all). |
| Medication adherence (6mfu only) |  |
| Medication knowledge | Subscale constructed from summing items I3-I5 from the Modified Morisky Scale (MMS). Yes (0) No (1). Item I5 is reverse scored. |
| Medication motivation | Subscale constructed from summing items I1, I2, I6 from the Modified Morisky Scale (MMS). Yes (0) No (1). |
| Social capital | Item H4 from the Health Survey for England measuring satisfaction with opportunities to participate in the community. Scores range from 1 (Strongly disagree) to 5 (Strongly agree). |
| Service use | Frequency of contact with Primary Care services (GP and nurse contact) and hospital outpatient visits in previous 6 months (O1-O6 and P1). |
| Social networks | Illness work measure: computed as the rating 0 (no help) to 4 (a lot of help). Network member scores are summed. For example, the score for a network with 3 people would range from 0 – 12 whereas a score for a network with 10 people would range from 0 - 40. |

**Statistical tests to be employed:**

Analysis of Secondary Outcomes

Two-level (patients within practices) multivariate regression

Dependent variable: Secondary outcome

Independent variables: Treatment group; awareness of CKD at baseline, baseline value of secondary outcome if available (if unavailable, baseline value for general health will be used as a proxy); design factors (age-group, smoking status and additional vascular disease). The analysis will also control for the clustering of patients within GP practices.

Level of significance: 0.05

Adjustment of significance and confidence levels due to multiplicity of outcomes

The analysis of secondary outcomes is regarded as exploratory and therefore not subjected to adjustment for multiple testing.

Alternative tests if distribution assumptions are violated

Regression with bootstrapping.

# Sensitivity analysis

See relevant section below

**5. Sensitivity analyses**

To assess sensitivity of the results to the missing values imputation, a second analysis will be conducted based on complete cases only.

**6. Treatment of missing data**

Multiple imputation (MI) using the chained equations approach will be used to generate 5 MI datasets and results will be combined across these to obtain a final result. The MI routine will use all measured variables, including those not used in the RCT analysis commands themselves (see Addendum), at both baseline and follow-up to generate full datasets for all patients returning at least one questionnaire.

**7. Distributional tests**

We will examine the distributional properties of each outcome variable. Variables for which skewness or kurtosis >1.5 or non-normal will be analysed using a bootstrap method.

**8. Bootstrapping**

Bootstrapping of p-values and CIs will be applied for outcome variables with skew or kurtosis>1.5. In these cases the bootstrapped estimate of standard error will be used. Prior to any bootstrapping a set of pseudorandom numbers will be generated (depending upon how many outcomes have skew or kurtosis >1.5 or are not normally distributed) using random.org to act as seeds for each bootstrap analysis.

**9. Choice of covariates**

The covariates to be included in all primary and secondary analyses will be selected in the below manner.

First, the baseline values of the outcome that is the focus of each analysis, when available (General health score will be used as a proxy when baseline value unavailable). Second, the below set of pre-specified covariates will automatically be included.

| **Pre-specified covariates** | **Description** |
| --- | --- |
| Awareness of CKD at baseline | Has patient has ticked ‘yes’ to ‘kidney problems in section A1g. |
| GP practice | Patient’s GP practice |
| Age | Question L2  Recoded as <75, 75+. |
| Additional vascular disease | Question A1  Self-reported at least one from: Angina or heart attack, Irregular heartbeat, stroke, PVD, heart failure. Coded as (0) No and (1) Yes |
| Smoking status | Question F7  Coded as (0) Non smoker or (1) Smoker |

**Addendum: List of all variables**

Primary outcomes

| **Primary Outcome (all at 6 months)** | **Description** |
| --- | --- |
| Blood pressure control | Computed as a dichotomy (controlled/uncontrolled) based upon the NICE guidelines: <140/90 for patients without proteinuria and <130/80 for patients with proteinuria). (0) Uncontrolled (1) Controlled. |
| Self-management ability (HeiQ – positive and active engagement in life) | Score on the ‘positive and active engagement in life’ subscale from the HeiQ (Health education and impact questionnaire) scale, computed as the mean across the 5 items, rescaled to the range 0-100. Items D1, D4, D7, D8, D12. |
| Health-related Quality of Life | The HRQOL weight derived from the ED-5D  Items A1 – A5 |

Secondary outcomes

| **Secondary Outcome** | **Description** | |
| --- | --- | --- |
| **Outcomes at 6 months** | | |
| Self management (heiQ) | | Computed as the mean across the items for each subscale, rescaled to the range 0-100. Items E1 – E31. |
| Social integration and support | |
| Skills and technique acquisition | |
| Emotional wellbeing | |
| Self-monitoring and insight | |
| Health services navigation | |
| Self-care activity | | Summary of Diabetes Self-Care Activities (SDSCA). Computed as the sum of items F1 – F7 rescaled to the range 0 -7. |
| General anxiety | | Anxiety subscale from the Hospital Anxiety and Depression Scale (HADS-A). Computed as the sum of items G1-G7. |
| CKD specific anxiety (6mfu only) | | One item - C2, ‘How much do your Kidney Problems (CKD) affect you emotionally? With a range of 0 (not affected at all) – 10 (extremely affected). |
| General Health | | Question C1  Treated as a continuous outcome. Five levels, recoded as (1) Poor (2) Fair (3) Good (4) Very good and (5) Excellent. |
| Social/Role Activities Limitations score | | Scale constructed from Questions C2-C5 as documented by Lorig  Overall score scaled to range from 0-100. |
| Energy/Vitality score | | Scale constructed from Questions C6-C10 as documented by Lorig.  Overall score is scaled to range from 0 to 100 |
| Psychological Wellbeing | | Scale constructed from Questions G1-G5 as documented by Lorig  Overall score scaled to range from 0-100 |
| Loneliness | | UCLA Loneliness Scale items J1 – J9. Scores range from 9 (very lonely) to 36 (Not lonely at all). |
| Medication adherence (6mfu only) | |  |
| Medication knowledge | | Subscale constructed from summing items I3-I5 from the Modified Morisky Scale (MMS). Yes (0) No (1). Item I5 is reverse scored. |
| Medication motivation | | Subscale constructed from summing items I1, I2, I6 from the Modified Morisky Scale (MMS). Yes (0) No (1). |
| Social capital | | Item H4 from the Health Survey for England measuring satisfaction with opportunities to participate in the community. Scores range from 1 (Strongly disagree) to 5 (Strongly agree). |
| Service use | | Frequency of contact with Primary Care services (GP and nurse contact) and hospital outpatient visits in previous 6 months (O1-O6 and P1). |
| Social networks | | Illness work measure: computed as the rating 0 (no help) to 4 (a lot of help). Network member scores are summed. For example, the score for a network with 3 people would range from 0 – 12 whereas a score for a network with 10 people would range from 0 - 40. |

Pre-specified covariates

| **Pre-specified covariates** | **Description** |
| --- | --- |
| Awareness of CKD at baseline | Has patient ticked ‘yes’ to ‘kidney problems in section A1G. |
| GP practice | Patient’s GP practice |
| Age | Question L2  Recoded as <75, 75+. |
| Additional vascular disease | Question A1  Self-reported at least one from: Angina or heart attack, Irregular heartbeat, stroke, PVD, heart failure. Coded as (0) No and (1) Yes |
| Smoking status | Question F7  Coded as (0) Non smoker or (1) Smoker |

Other possible covariates collected but not included in the analysis at present (list is not exhaustive).

| **Additional covariates** | **Description** |
| --- | --- |
| Normalisation | Items I1-I4. |
| Employment status | Section M1: Eight categories, including In Paid Work, Unemployed, Retired, etc |
| Social activities, services and groups | Items R1 – R2 and H3. |
| Community | Items H2a – H2f. |
| Trust and helpfulness | Items H5-H6. |
| Health related internet use | Section L9. |
| Subjective social status ladder | Item U1. |
| Living situation | Section L3: Seven categories, recoded as Lives Alone versus Lives with others. |
| Accommodation | Section L5. (1) Owner occupied mortgaged, (2) Rented local authority, (3) Rented privately (4) Other. |
| Highest qualification | Section L7. |
| Household income | Section N1: From Nil up to £52,000 or more per year |
| Resource generator | Section T1. |
